# Association of a *Pre-miR-1453* Polymorphism with Growth Traits and Serum Biochemical Parameters in an F_2_ Chicken Population

**DOI:** 10.3390/ani16152394

**Published:** 2026-08-03

**Authors:** Jianzhou Shi, Lunguang Yao, Guirong Sun

**Affiliations:** 1 The Shennong Laboratory, Zhengzhou 450046, China; shijian1109@163.com; 2 School of Life Science, Nanyang Normal University, Nanyang 473061, China; lunguangyao@163.com; 3 College of Animal Science and Technology, Henan Agricultural University, Zhengzhou 450002, China

**Keywords:** microRNAs (miRNAs), single-nucleotide polymorphism (SNP), F_2_ resource population, *pre-miRNA-1453*, growth performance, carcass traits, serum biochemical parameters

## Abstract

MicroRNAs (miRNAs) serve as pivotal post-transcriptional modulators of gene expression that govern animal growth and development processes. The present study aimed to investigate the relationship between a single-nucleotide polymorphism (SNP) located within the *pre-miR-1453* precursor and growth traits, carcass traits, body size traits, and serum biochemical parameters in an F_2_ chicken resource population derived from Gushi and Anka crosses (*n* = 860). Genotyping of the rs16159272 polymorphism (+31 bp C > T) was performed using MALDI-TOF mass spectrometry, and population genetic parameters, secondary structure prediction, and association analyses were conducted. The results showed that the SNP was moderately polymorphic (PIC = 0.3174) and significantly associated with hatch weight (*p* < 0.01; CT > TT > CC), as well as body weight at 2 and 4 weeks of age (*p* < 0.05; TT > CT > CC), but not with carcass or body size traits (*p* > 0.05). For serum biochemical parameters, total protein, cholinesterase, and creatine phosphokinase showed extremely significant differences across genotypes (*p* < 0.01), whereas albumin, globulin, and lactate dehydrogenase showed significant differences (*p* < 0.05). Structural prediction revealed that the C > T mutation increased the minimum free energy by 3.8 kcal/mol, reducing the stability of the *pre-miR-1453* stem-loop. Target gene prediction and enrichment analysis indicated that *pre-miR-1453* may participate in the regulation of chicken growth and serum biochemical parameters through pathways related to neuron development, cytoskeletal dynamics, and energy metabolism. These findings suggest that the *pre-miR-1453* rs16159272 polymorphism could act as a candidate genetic indicator for marker-aided breeding in poultry.

## Simple Summary

Background: MicroRNAs are small molecules that help control how genes work in animals, and they play important roles in growth and development. This study investigated a specific genetic change (single-nucleotide polymorphism, SNP) in a microRNA called *miR-1453* and its relationship with growth and health traits in chickens. Method: A total of 860 chickens from a special breeding population were examined; this population was created by crossing slow-growing Gushi chickens with fast-growing Anka broilers. Result: The results showed that this genetic change was linked to differences in hatch weight, as well as body weight measured at 2 and 4 weeks post-hatching, alongside altered levels of specific serum proteins and enzymes. The genetic change also affected the shape and stability of the *miR-1453* molecule. Target gene prediction and pathway analysis suggested that *miR-1453* may influence chicken growth through pathways involved in nerve development, cell structure, and energy use. Conclusions: These results indicate that this genetic marker could be useful for selecting chickens with better growth performance in breeding programs.

## 1. Introduction

Poultry production plays a pivotal role in global animal agriculture, significantly contributing to the development of the livestock economy [1,2]. Growth performance traits in chickens, including daily weight gain, slaughter weight, and feed conversion efficiency, directly determine production costs [3,4], while enzyme activities, such as alkaline phosphatase and aspartate aminotransferase, serve as critical indicators reflecting metabolic status and health levels [5,6,7]. Improving chicken growth performance efficiently has become a primary objective of the poultry breeding industry and represents a core strategy for promoting the large-scale upgrading of the poultry sector [8,9,10]. With the advancement of molecular biology technologies, genetic improvement has entered the molecular era [11].

Research on non-coding RNAs, particularly microRNAs (miRNAs), has provided novel insights into understanding the molecular regulatory mechanisms underlying reproductive, production, and meat quality traits in poultry. MiRNAs constitute a group of endogenous, small non-coding RNA molecules roughly 22 nucleotides long. They modulate gene expression post-transcriptionally via interaction with the 3′ untranslated region (3′ UTR) of mRNAs to inhibit translation and/or promote mRNA degradation, thereby exerting significant effects on core physiological processes including cell proliferation, differentiation, and metabolic homeostasis, and playing crucial roles in various biological processes [12,13,14]. An in-depth understanding of miRNA biogenesis, function, and their association with livestock traits is essential for promoting genetic improvement and enhancing production efficiency in animal husbandry [15]. SNPs, constituting the predominant type within genomic variation, can affect the structure and function of miRNA genes and subsequently regulate target gene expression. SNPs present in miRNA genes may influence precursor miRNA processing or the binding to target mRNAs, thereby resulting in altered target gene abundance and phenotypic variation [16]. SNPs play important roles in the generation, processing, and functional regulation of miRNAs and are associated with various phenotypes and disease occurrence [14]. In the past several years, a number of research works have verified that the biogenesis and function of miRNAs are regulated by SNPs, which are widely distributed in genomic regions, including miRNA genes themselves and the regulatory sequences of their target genes [17,18]. SNPs have found increasing application in dissecting the genomic foundation of economically important traits in farm animals and poultry. Research has confirmed that *miR-301a-5p* participates in male germ cell development through direct targeting of *TGFβ2*, a pivotal component of the *TGF-β* cascade, providing novel understanding of the mechanistic principles whereby this miRNA modulates chicken germ cell maturation and sperm production [19]. Additionally, miRNA expression in turkey muscle changed significantly under different heat stress conditions, indicating that miRNAs are of great significance in regulating cell proliferation and differentiation [20]. Furthermore, *miR-181a-5p* exerts suppressive effects on *VNN1* levels in chicken hepatocytes, suggesting that miRNAs participate in the regulation of signaling pathways closely associated with energy metabolism and growth [21]. It has also been found that *miR-148a-3p* plays an important regulatory role in chicken breast muscle development by targeting the *DYNLL2* gene, thereby promoting the expression of myosin heavy-chain proteins and myoblast differentiation [22]. SNPs not only provide an important genetic basis for the growth, development, and economic traits of livestock and poultry, but also offer powerful tools for molecular breeding practice. Our previous work on *pre-miR-3528* also verified that SNP rs14098602 (+12 bp A > G) within the precursor region significantly correlated with early growth performance, carcass characteristics, meat quality and multiple serum biochemical parameters in the same Gushi–Anka F_2_ resource population, and this variant was proposed as a feasible molecular marker for chicken marker-assisted breeding [23]. However, the association between genetic variations in *miR-1453* and economic traits in livestock remains largely unexplored.

Preliminary screening of the Gushi–Anka F_2_ resource population revealed that *pre-miR-1453* harbors polymorphic variation in its precursor region, providing a genetic basis for molecular marker development; however, the association between *miR-1453* and economic traits in chickens remains unexplored. Therefore, miR-1453 was selected as a candidate gene for association analysis with growth and serum biochemical parameters in this study.

Here, we utilized an F_2_ chicken reference population derived from crossing Gushi chickens and Anka chickens to screen for SNPs within *miR-1453* and conduct association analyses across a range of phenotypic measurements, covering growth and developmental traits, carcass characteristics, and blood serum biochemical indices. The predicted target genes underwent GO and KEGG pathway enrichment assessments. Our aim was to evaluate these detected miR-SNPs as candidate markers for enhancing economically valuable traits in chickens, thereby offering a theoretical basis for poultry genetic breeding and providing useful guidance for subsequent genetic optimization plans.

## 2. Materials and Methods

### 2.1. Study Population

Experimental birds (*n* = 860) were sourced from the Gushi–Anka F_2_ reference pedigree originally described by Shi et al. [24,25]. This pedigree was derived from reciprocal crosses between Gushi (slow-growing, Chinese indigenous) and Anka (fast-growing). Selection criteria included breed conformation, egg production performance, optimal body mass, and authenticated lineage. F_0_ matings followed a 1:6 male-to-female ratio to maximize heterozygosity in the F_1_ progeny. For F_2_ production, one F_1_ cockerel per family was selected from the F_1_ progeny of each family and mated to hens from unrelated families at a 1:9 sire-to-dam ratio, yielding 7 F_1_ sires and 63 F_1_ dams in total. The F_2_ generation was structured into 7 families (A–G): four derived from Anka sires mated to Gushi dams, and 3 from Gushi sires mated to Anka dams. These matings produced 860 F_2_ offspring, which were housed in identical cage systems under standardized husbandry with ad libitum access to feed and water until 12 weeks of age. At 84 days after hatching, all birds were sampled following approved ethical protocols.

### 2.2. Determination of Growth and Carcass Traits

Individual body weight (BW) was recorded upon hatching (0W) and subsequently at 2W, 4W, 6W, 8W, 10W, and 12W post-hatch (the latter corresponding to the slaughter age).

For body size traits, we recorded a series of morphometric indices at predetermined developmental time points: measurements of the appendicular skeleton (shank length, SL; shank girth, SG), the thoracic cage (chest depth, CD; chest breadth, CB; breast-bone length, BBL; pectoral angle, PA), and trunk dimensions (body slanting length, BSL; pelvis breadth, PB).

For carcass traits, we quantified the following parameters post-slaughter: carcass weight (CW); semi-evisceration weight (SEW), determined after removing the trachea, esophagus, crop, gastrointestinal tract, gallbladder, pancreas, spleen, and reproductive organs from the carcass; evisceration weight (EW), calculated by further excluding the head, feet, heart, liver, proventriculus, gizzard, and abdominal fat from SEW; breast muscle weight (BMW) and leg muscle weight (LMW). These analytical procedures were detailed in Han et al. [26].

### 2.3. Determination of Serum Biochemical Parameters

Venous blood specimens were drawn from the jugular vein of all 860 F_2_ chickens at 84 d during slaughter and placed into anticoagulant-free centrifuge tubes. Blood-containing centrifuge tubes were incubated at an oblique angle at ambient temperature for 1 h to facilitate serum preparation. The tubes were thereafter spun at 3000 rpm for 15 min at 4 °C. The harvested serum was aliquoted and preserved at −80 °C pending serum biochemical assessment.

A panel of 17 serum biochemical parameters was evaluated, categorized by physiological function as follows: (i) hepatobiliary function and synthetic capacity markers—alanine aminotransferase (ALT), aspartate aminotransferase (AST), alkaline phosphatase (AKP), γ-glutamyl transpeptidase (γ-GT), and cholinesterase (CHE); (ii) muscular and cellular energy metabolism indicators—creatine phosphokinase (CPK) and lactate dehydrogenase (LDH); (iii) pancreatic exocrine function and carbohydrate metabolism—amylase (AMY) and glucose (GLU); (iv) protein nutritional status—total protein (TP), albumin (ALB), and globulin (GLOB); (v) renal filtration function—creatinine (CRE); and (vi) lipid metabolism and cardiovascular risk profile—total cholesterol (TC), triglycerides (TGs), high-density lipoprotein (HDL), and low-density lipoprotein (LDL). All assays were performed using commercial kits (Jiancheng Bio, Nanjing, China) according to the manufacturer’s protocols. The specific analytical procedures were detailed in Han et al. [27].

### 2.4. Polymorphism Screening

Venous whole blood containing anticoagulant ethylenediaminetetraacetic acid (EDTA) was used to extract DNA from 860 F_2_ birds of the bird resource population by the standard phenol–chloroform extraction coupled with a commercially available DNA purification kit (Tiangen Biotech, Beijing, China). MALDI-TOF MS-based genotyping was performed [28], and each DNA sample’s concentration was precisely quantified following the manufacturer’s protocol. All samples were subdivided and cryopreserved at −80 °C until further use. A total of 100 F_2_ chicken genomic DNA templates, chosen at random, were normalized to identical working concentrations and subsequently combined in equal volumes to generate a pooled sample. The genomic sequence of chicken *miR-1453* (miRBase: MI0007058; RefSeq: NR_035020.1; genomic coordinates: 1424639…1424712 on chromosome 20, negative strand) was retrieved from miRBase. PCR primers targeting the *miR-1453* precursor region (forward primer: 5′-CTGTCTCCATCCTCTGGGCGCTTC-3′; reverse primer: 5′-ACCACCAATAACAGCAGCTATCTG-3′) were designed for SNP detection. Genome-wide polymorphism screening of the pooled DNA from the F_2_ birds was conducted by Sangon Biotech (Shanghai, China).

### 2.5. Genotyping

Genomic DNA was subjected to SNP genotyping via MALDI-TOF MS at an accredited commercial laboratory. Within the F_2_ chicken resource population, the rs16159272 SNP (+31 bp C > T) situated in *pre-miR-1453* was genotyped on the MassArray-iPLEX GOLD system (Sequenom Inc., San Diego, CA, USA). For this purpose, amplification was carried out with designated primer pairs, followed by single-base extension using a specific primer; both primer sets were constructed with Professional Assay Design software (v3.1). The following primers were utilized: a forward primer for the *pre-miR-1453* SNP (5′-ACGTTGGATGTAGAGAGAGTCTGGTGTGTG-3′), a reverse PCR amplification primer for the *pre-miR-1453* SNP (5′-ACGTTGGATGATAACTGGACACTGTGGGAG-3′), and a single-base extension (SBE) primer (5′-GGCTAGCACCATGCAGTACC-3′). Genotyping of the *pre-miR-1453* SNP was conducted by MALDI-TOF MS in accordance with the standard operating procedure. Allele peak area and SNP call rate metrics were recorded from the MassArray output. Genotype calling was performed automatically via the proprietary Assay Design 3.1 software.

Population genetic parameters of the *miR-1453* (+31 bp C > T) mutation site were analyzed, including polymorphism information content (PIC), observed heterozygosity (H_O_), and effective number of alleles (Ne).

### 2.6. Prediction of Pre-miR-1453 Secondary Structure

The optimal secondary structure (lowest free energy) of chicken *pre-miR-1453* carrying the rs16159272 SNP (C > T) was predicted using the M-fold online tool (http://www.unafold.org/mfold/applications/rna-folding-form-v2.php, retrieved on 6 June 2025). This difference in free energy between the C and T alleles of *pre-miR-1453* (*Gallus gallus*) was calculated to evaluate the impact of this allelic pair on *pre-miR-1453* structural stability.

### 2.7. Data Statistical Analyses

Correlations between the *pre-miR-1453* SNP and growth traits, developmental characteristics, and biochemical parameters in the Gushi × Anka F_2_ resource population were evaluated using SPSS 20.0. A linear mixed model in SPSS was employed to examine the relationships between genetic variants and production traits within this F_2_ population. A pair of linear mixed-effects models were fitted to examine the relationships between the polymorphism and economic traits in the F_2_ population. Model I was applied to analyze growth, developmental, and biochemical characteristics, whereas Model II was employed for carcass traits with carcass weight included as a covariate to account for body weight influence. Multiple comparisons for significant genotype effects were adjusted using the Bonferroni correction. The model specifications are detailed below:

Model I: Y_ijkl_ = μ + G_i_ + S_j_ + H_k_ + f_l_ + e_ijklm_

Model II: Y_ijkl_ = μ + G_i_ + S_j_ + H_k_ + f_l_ + b (W_ijklm_ − W) + e_ijklm_

Y_ijkl_ denotes observed phenotypic trait value; μ represents overall population mean; G_i_ indicates the fixed effect of genotype with i indexing three categories (CC, CT, and TT); f_l_ captures the random effect of family where l corresponds to seven reference families; s_j_ reflects the fixed effect of sex with j spanning two levels; H_k_ signifies the fixed effect of hatch across k = two levels; b stands for regression coefficient associated with carcass weight; W_ijklm_ refers to individual carcass weight; W designates mean carcass weight; and e_ijklm_ constitutes a random error term [23]. The significance threshold for fixed effects was set at *p* < 0.05.

### 2.8. Target Gene Prediction and Enrichment Analysis of miR-1453

The potential target genes of *pre-miR-1453* were predicted based on two online databases, TargetScan (https://www.targetscan.org/vert_80/, accessed on 6 June 2025) and miRDB (https://mirdb.org/). To reduce false positives and enhance prediction reliability, the intersection of target genes from both databases was used as the candidate target genes. A Venn diagram was generated using the web-based tool Venny (https://bioinfogp.cnb.csic.es/tools/venny/index.html, accessed on 6 June 2025). Thereafter, enrichment analyses were performed on the candidate target genes using the OmicShare bioinformatics cloud platform (https://www.omicshare.com). The analyses included GO and KEGG pathway enrichment analyses.

## 3. Results

### 3.1. Detection of Pre-miRNA-1453 Gene Polymorphism

**Figure 1:**
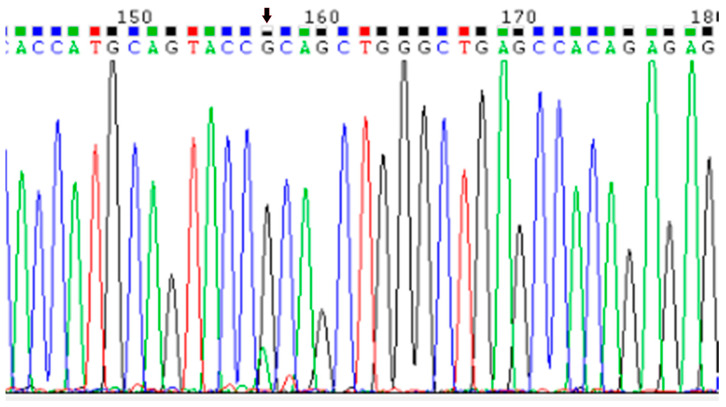
Partial sequencing chromatogram of the *pre-miR-1453* region. Arrow: SNP rs16159272 (*pre-miR-1453* + 31bp C > T; G > A on the plus strand) at position 157 bp.

To identify polymorphisms in *pre-miR-1453*, PCR-purified products were subjected to sequencing analysis. A G/A heterozygous peak was observed at position 157 bp in the sequencing chromatogram (Figure 1), which corresponds to the reverse-complement base (G) of the 31st nucleotide (C) in the NCBI reference sequence NR_035020.1. This indicates a C/T polymorphism at the 31st position of the *miR-1453* precursor, i.e., a C > T mutation at position +31 bp in *pre-miR-1453*.

### 3.2. Genotyping of the Pre-miR-1453 SNP

**Figure 2:**
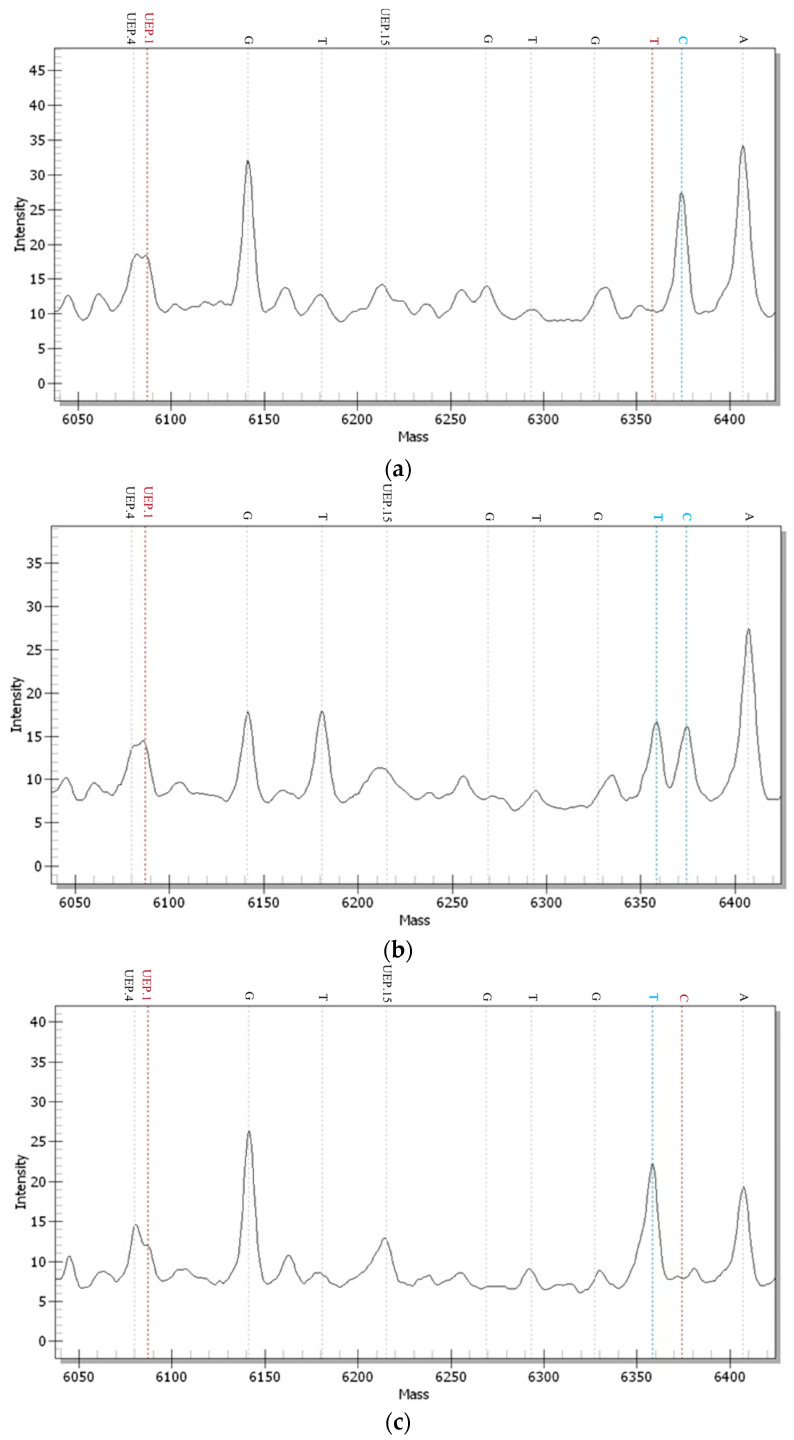
Representative mass spectra of distinct *pre-miR-1453* genotypes. (**a**) CC genotype; (**b**) CT genotype; (**c**) TT genotype. Dashed lines of different colors indicate expected allele peak positions annotated by the MassArray software v5.0.1.

Figure 2 presents the mass spectrometry profiles of the three variant genotypes (CC, CT, and TT). In the F_2_ generation chicken resource population, the allele frequencies of the *pre-miR-1453* SNP were 0.7283 (C) and 0.2717 (T), whereas the genotype frequencies were 0.5921 (CC), 0.2725 (CT), and 0.1355 (TT), respectively.

The PIC, H_O_, and Ne of the *miR-1453* (+31 bp C > T) mutation site were 0.3174, 0.3957, and 1.6549, respectively. According to the criterion that a locus with 0.25 < PIC < 0.5 falls into the category of moderate polymorphism, and given that this site exhibited three genotypes (CC, CT, and TT), it was considered suitable for association analysis with relevant economic traits in the resource population.

### 3.3. Predicted Secondary Structure of Pre-miR-1453

**Figure 3:**
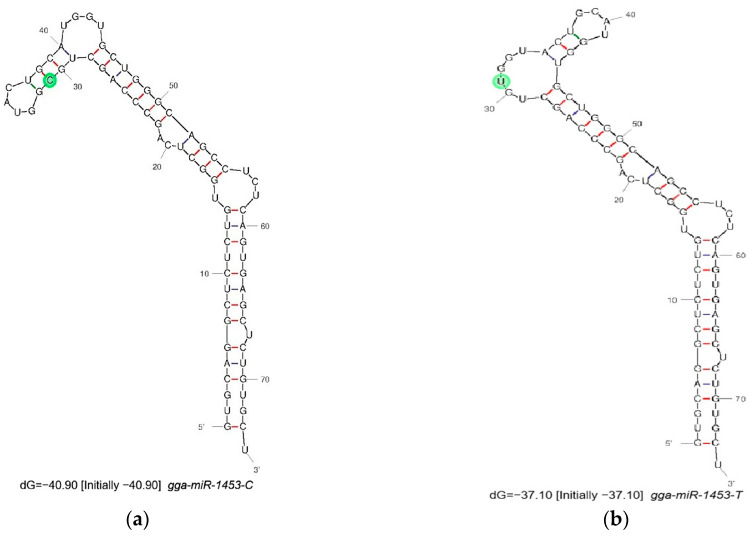
Secondary structure prediction of different alleles (C/T) of *pre*-*miRNA-1453*. (**a**) Wild type; (**b**) mutant type. The green circle indicates the +31 bp mutation site.

M-fold was used to predict secondary structural alterations in *pre-miR-1453* RNA resulting from SNP allelic differences. Secondary structure prediction revealed that the rs16159272 C > T transition at the +31 bp site of *pre-miR-1453* markedly modified its stem-loop configuration and elevated the lowest free energy by 3.8 kcal/mol (Figure 3). The rs16159272 (C/T) polymorphism reduced the stability of the secondary structure of *pre-miR-1453*.

### 3.4. Pre-miR-1453 Variant and Phenotypic Traits

In this study, the *pre-miR-1453* genotypes (CC, CT, and TT) in the F_2_ chicken resource population were subjected to association analysis with carcass traits, and the results are presented in Table 1. No significant associations were evident between the rs16159272 variant and any carcass traits (*p* > 0.05). Among the genotypes, the leg muscle weight (LMW) at 12 weeks of age exhibited the rank order CT > TT > CC; the breast muscle weight (BMW) followed the order CC > CT > TT; and the semi-evisceration weight (SEW), carcass weight (CW), and evisceration weight (EW) all showed the order CT > CC > TT.

An association analysis was performed between the *pre-miR-1453* genotypes (CC, CT, and TT) and growth traits in the F_2_ chicken population, and the results are presented in Table 1. The results showed that the SNP (rs16159272) in the *pre-miRNA-1453* gene was significantly linked to hatch weight (0BW) (*p* < 0.01), with the 0BW of different genotypes following the rank order CT > TT > CC. The SNP (rs16159272) was also significantly linked to body weight at 2 weeks of age (2BW) and 4 weeks of age (4BW) (*p* < 0.05), both showing the rank order TT > CT > CC. Furthermore, at 6 and 10 weeks of age, individuals with the CT genotype exhibited higher body weight than those with the TT and CC genotypes (CT > TT > CC), whereas the rank order was TT > CC > CT for 8BW and CT > CC > TT for 12BW.

This study performed an association analysis between the rs16159272 of the *pre-miR-1453* gene and body size traits in chickens, and the results are shown in Table 2. Data analysis revealed that compared with individuals carrying the CT or TT genotype, individuals with the CC genotype exhibited higher phenotypic values for the following traits (CC > CT > TT): 0SL and 8-week (SG, PA, BSL). This was also the case for the following traits (CC > TT > CT): 8-week (CD, BBL, CB, PB). Compared with individuals carrying the CC or TT genotype, individuals with the CT genotype exhibited lower phenotypic values for the following traits (TT > CC > CT): 4-week (SL, BSL, PB), 8SL, and 12-week (SL, BBL, PA, BSL, PB). This was also the case for 12SG (CC > CT, and TT > CT). Compared with individuals carrying the CT or CC genotype, individuals with the TT genotype exhibited higher phenotypic values for the following traits (TT > CT > CC): 4-week (SG, CD, PA, BBL, CB). Compared with individuals carrying the TT or CC genotype, individuals with the CT genotype exhibited higher phenotypic values for the following traits (CT > CC > TT): 12-week (CD, CB).

Correlation analysis was performed between the different genotypes (TT, CC, and CT) of the *pre-miR-1453* SNP and serum biochemical parameters in the F_2_ resource population, and the results are provided in Table 3. The analysis disclosed the following findings: TP, CHE, and CPK showed extremely significant differences among different genotypes (*p* < 0.01). For TP and CHE, the TT genotype was significantly higher than the CC and CT genotypes, with the CT genotype being the lowest (TT > CC > CT). For CPK, the CC genotype was significantly higher than the CT and TT genotypes, with the TT genotype being the lowest (CC > CT > TT).

ALB, GLOB, and LDH showed significant differences among different genotypes (*p* < 0.05). For ALB and GLOB, the TT genotype was significantly higher than the CC and CT genotypes, with the CT genotype being the lowest (TT > CC > CT). For LDH, the CC genotype was significantly higher than the CT and TT genotypes, with the TT genotype being the lowest (CC > CT > TT).

No significant differences were observed for other biochemical parameters among different genotypes (*p* > 0.05). Among these, ALT, AST, TC, TG, and HDL showed the highest values in the CC genotype, followed by the CT genotype, with the TT genotype being the lowest (CC > CT > TT); γ-GT, AMY, and GLU showed the highest values in the CT genotype, followed by the CC genotype, with the TT genotype being the lowest (CT > CC > TT); AKP showed the highest values in the TT genotype, followed by the CT genotype, with the CC genotype being the lowest (TT > CT > CC); CRE and LDL showed the highest values in the TT genotype, followed by the CC genotype, with the CT genotype being the lowest (TT > CC > CT).

### 3.5. Target Gene Prediction and Enrichment Analysis Results of miR-1453

The target genes of *pre-miR-1453* were predicted using the TargetScan and miRDB online databases, yielding 971 and 703 predicted targets, respectively. To reduce potential false positives and enhance reliability, the intersection of predictions from both databases was extracted, resulting in 153 common target genes.

**Figure 4:**
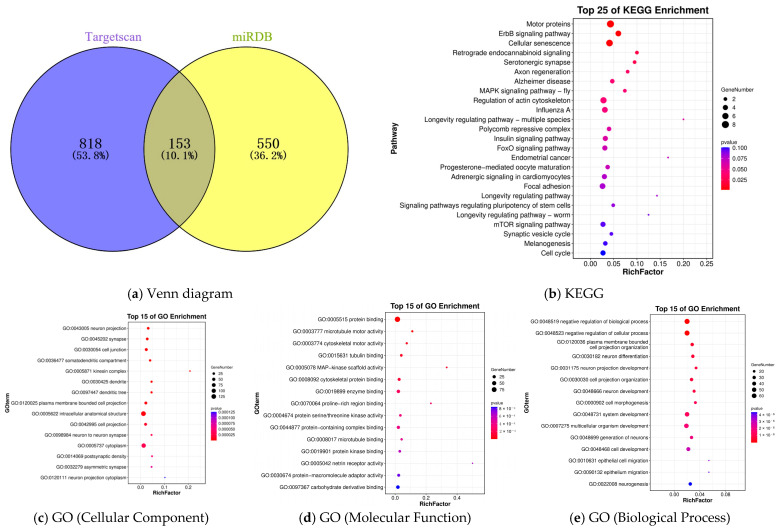
Target gene prediction and enrichment analysis of *miR-1453*.

Based on these predicted candidate target genes, a Venn diagram was constructed (Figure 4a). Enrichment analyses covering GO and KEGG pathways were performed on the 153 candidate target genes of *pre-miR-1453* using OmicShare tools. GO enrichment analysis was categorized into three domains, namely Cellular Component (CC), Molecular Function (MF), and Biological Process (BP), with the respective results illustrated in Figure 4c–e. The top 15 enriched terms in each of the BP, MF, and CC categories were selected for annotation, with *p* < 0.05 as the significance threshold. KEGG pathway enrichment analysis was subsequently performed on the intersecting target genes (Figure 4b). The results showed that BP was mainly enriched in neuron differentiation and development, cell projection organization, negative regulation of biological processes, and epithelial cell migration. CC was primarily localized to neuron projections, synapses, dendrites, the somatodendritic compartment, and the cytoplasm. MF was significantly enriched in protein binding, microtubule motor activity, cytoskeletal protein binding, kinase activity, and netrin receptor activity. A KEGG pathway enrichment analysis demonstrated that these candidate target genes exhibited significant enrichment in motor proteins, the ErbB signaling pathway, cellular senescence, retrograde endocannabinoid signaling, the serotonergic synapse, axon regeneration, the MAPK signaling pathway, regulation of the actin cytoskeleton, and the insulin/FoxO/mTOR signaling pathways. These results suggest that *pre-miR-1453* may participate in the regulation of chicken growth and serum biochemical parameters by modulating neuron development, synaptic function, cytoskeletal dynamics, and energy metabolism-related pathways.

## 4. Discussion

In this study, SNP rs16159272 (+31 bp C > T) was identified in the precursor region of *pre-miR-1453* in a Gushi × Anka F_2_ chicken resource population. This locus was moderately polymorphic (PIC = 0.3174) and suitable for association analysis. M-fold prediction revealed that the C > T mutation increased the minimum free energy by 3.8 kcal/mol, reducing the stability of the stem-loop structure. This finding is consistent with previous studies conducted in the same F_2_ population: rs16681031 (+60 bp C > G) in *pre-miR-1658* [24], rs15179830 (+90 bp T > G) in *pre-miR-1687* [25], and rs313467992 (+35 bp C > A) in *pre-miR-1606* [26] all significantly altered secondary structure stability, whereas our previous study on *pre-miR-3528* rs14098602 (+12 bp A > G) did not significantly alter the structure [23], suggesting that the functional effects of SNPs in miRNA precursor regions are locus-specific. Alterations in structural stability may affect the cleavage efficiency of Drosha and Dicer, thereby altering the expression level of mature *miR-1453*.

rs16159272 was significantly associated with hatch weight (*p* < 0.01, CT > TT > CC), as well as body weight at 2 and 4 weeks of age (*p* < 0.05, TT > CT > CC), but showed no significant association with body weight after 6 weeks of age or carcass traits (*p* > 0.05), indicating that *miR-1453* primarily regulates early growth. Comparison with other miRNA-SNPs in the same population revealed stage-specific regulatory differences: *miR-1658* [24] affected body weight at 6–12 weeks of age (late growth), complementary to the early growth effect (0–4 weeks) observed in this study; *miR-1687* [25] affected body weight at 0–10 weeks of age, with a broader time window; *miR-1606* [26] affected body weight at 10–12 weeks of age (late growth), unlike *miR-1453*; and *miR-3528* [23] affected body weight at 0–4 weeks of age and was associated with evisceration weight, whereas *miR-1453* did not affect carcass traits. This developmental stage specificity may be attributed to the functional characteristics of *miR-1453* target genes. Target gene prediction revealed that 153 common target genes were significantly enriched in pathways related to neuron differentiation, cytoskeletal dynamics, and insulin/FoxO/mTOR energy metabolism, rather than direct muscle growth pathways. It should be noted that hatch weight (0BW) may be influenced by maternal effects, including egg weight and incubation conditions. Egg weight data were not recorded in this study, which is acknowledged as one of the limitations. However, the F_2_ resource population was generated through random mating of F_1_ individuals, thereby minimizing systematic maternal genotype effects. Furthermore, the genotype effects remained significant at 2 and 4 weeks of age, when maternal effects have largely diminished, supporting the authenticity of genetic effects rather than purely maternal environmental influences. Future studies should consider recording egg weight as a covariate to further clarify the relative contributions of genetic and maternal effects on early growth traits.

Rs16159272 was significantly associated with TP, CHE, and CPK (*p* < 0.01), as well as ALB, GLOB, and LDH (*p* < 0.05). For TP, ALB, GLOB, and CHE, the order was TT > CC > CT, whereas for CPK, the order was CC > CT > TT (*p* < 0.01), and for LDH, both CC and TT were significantly higher than CT (*p* < 0.05). Comparison with *miR-3528* [23] revealed that both miRNAs were associated with TP, ALB, GLOB, CHE, and LDH, but the direction of effects differed. In *miR-3528*, the GG genotype manifested significantly lower serum protein levels than the AA genotype (GG < AG < AA), whereas for *miR-1453*, the order was TT > CC > CT. This difference may reflect that different miRNAs regulate metabolic pathways through distinct target gene networks. In the target gene enrichment analysis, kinase activity corresponds to CPK/LDH function, protein binding corresponds to TP/ALB transport function, and the serotonergic synapse pathway may explain the variation in CHE, while the insulin/FoxO/mTOR pathway serves as a central hub for energy metabolism integration.

SNPs located within miRNA genes and their target binding sites have the potential to alter the production of mature miRNAs and the modulation of their targets, thus contributing to various physiological traits and biological processes [29]. SNPs located in the pre-miRNA region can profoundly affect miRNA function by altering local secondary structure or critical sequences. The main mechanisms include: interfering with the precise cleavage of precursors by Drosha/Dicer enzymes, affecting the yield and sequence accuracy of mature miRNAs; altering thermodynamic stability, leading to guide strand selection errors during RISC loading; and ultimately weakening or altering the silencing efficacy of miRNAs on their target genes [30]. With in-depth research on the application of miRNA SNPs in livestock and poultry, it has been found that these genetic variations may be closely related to production performance, meat quality traits, and disease resistance, providing a theoretical basis for future breeding strategies. Through research and analysis of SNPs in miRNA genes, more precise molecular markers can be provided for genetic improvement in animal husbandry, thereby enhancing the economic traits of animals. It is important to acknowledge several limitations of this study. First, the findings were based on a single F_2_ resource population derived from Gushi and Anka chickens, and the generalizability of these results to other chicken breeds, particularly commercial broiler lines, remains to be validated. Second, the functional mechanisms by which rs16159272 affects *miR-1453* biogenesis and target gene regulation have not been experimentally verified, and further studies, such as dual-luciferase reporter assays and gene expression analyses, are necessary to confirm the causal relationship between this SNP and the observed phenotypic variations.

## 5. Conclusions

This study identified the *pre-miR-1453* rs16159272 polymorphism (+31 bp C > T) in a Gushi × Anka F_2_ chicken resource population (*n* = 860). The SNP significantly affected early growth traits (hatch weight and body weight at 2 and 4 weeks of age) and serum biochemical parameters (TP, CHE, CPK, ALB, GLOB, and LDH) by altering the stability of the pre-miRNA stem-loop structure. Its unique phenotypic effect profile, which is complementary to those of other miRNA-SNPs previously identified in the same population, suggests that rs16159272 may serve as a supplementary candidate molecular marker, particularly when combined with other miRNA-SNPs identified in the same population, for marker-assisted selection programs aimed at improving early growth performance and metabolic health in chickens.

## 6. Patents

A patent application related to the findings of this study has been filed (Chinese Patent Application No. 202510675046.1, filed on 23 May 2025, currently under substantive examination).

## Figures and Tables

**Table 1:** Genotypic effects of the *pre-miR-1453* (rs16159272) variant on growth traits and carcass traits.

Traits	Genotype			*p*
	CC	CT	TT	
SEW	1102.74 ± 8.45	1110.90 ± 12.25	1098.70 ± 16.76	1.000
EW	923.04 ± 7.22	931.39 ± 10.67	917.13 ± 14.49	0.899
BMW	71.53 ± 0.69	70.92 ± 1.08	69.46 ± 1.39	0.109
LMW	98.10 ± 0.89	100.05 ± 1.30	99.48 ± 1.90	0.161
CW	1187.97 ± 8.98	1196.67 ± 12.95	1185.15 ± 17.28	0.939
0BW	30.19 ± 0.13 b	31.54 ± 0.18 a	31.22 ± 0.24 a	0.000 **
2BW	121.45 ± 0.86 b	124.66 ± 1.26 a	125.51 ± 1.58 a	0.038 *
4BW	319.93 ± 2.10 b	325.40 ± 3.19 a	330.48 ± 4.39 a	0.026 *
6BW	559.15 ± 4.65	572.16 ± 7.34	569.87 ± 9.73	0.266
8BW	816.77 ± 6.22	816.49 ± 9.41	828.22 ± 12.76	0.075
10BW	1112.83 ± 7.86	1128.59 ± 12.08	1113.00 ± 16.76	0.671
12BW	1353.55 ± 9.79	1363.93 ± 14.49	1338.79 ± 19.16	0.674

CC (*n* = 509), CT (*n* = 234), TT (*n* = 117). Mean ± SE. SEW, semi-evisceration weight; EW, evisceration weight; BMW, breast muscle weight; LMW, leg muscle weight; CW, carcass weight. 0BW, 2BW, 4BW, 6BW, 8BW, 10BW and 12BW refer to body weight measured at 0 days (hatch) and 2, 4, 6, 8, 10 and 12 weeks, respectively. Unit: g. Different letters within a row indicate significant differences (*p* < 0.05), while the same or no letters indicate no significant difference (*p* > 0.05). ** *p* < 0.01, * *p* < 0.05. The same as below.

**Table 2:** Genotypic effects of the *pre-miR-1453* (rs16159272) variant on body size traits.

Traits	Genotype			*p*
	CC	CT	TT	
0SL	2.598 ± 0.020	2.576 ± 0.008	2.582 ± 0.012	0.723
4SL	5.483 ± 0.042	5.451 ± 0.064	5.644 ± 0.088	0.204
8SL	7.942 ± 0.048	7.858 ± 0.066	7.951 ± 0.095	0.562
12SL	9.370 ± 0.044	9.330 ± 0.065	9.380 ± 0.093	0.808
4SG	2.680 ± 0.011	2.697 ± 0.015	2.730 ± 0.024	0.141
8SG	3.417 ± 0.013	3.411 ± 0.022	3.412 ± 0.031	0.971
12SG	3.840 ± 0.016	3.830 ± 0.024	3.840 ± 0.032	0.975
4CD	4.837 ± 0.033	4.865 ± 0.049	4.876 ± 0.074	0.829
8CD	6.563 ± 0.048	6.547 ± 0.064	6.398 ± 0.095	0.300
12CD	7.860 ± 0.039	7.950 ± 0.059	7.820 ± 0.076	0.373
4CB	4.063 ± 0.024	4.104 ± 0.035	4.146 ± 0.063	0.313
8CB	5.703 ± 0.031	5.676 ± 0.043	5.641 ± 0.059	0.647
12CB	6.317 ± 0.033	6.386 ± 0.048	6.280 ± 0.080	0.380
4BBL	6.199 ± 0.027	6.223 ± 0.039	6.234 ± 0.053	0.796
8BBL	8.928 ± 0.038	8.906 ± 0.057	8.831 ± 0.089	0.552
12BBL	11.002 ± 0.040	10.957 ± 0.062	11.008 ± 0.086	0.807
4PA	73.960 ± 0.257	74.140 ± 0.293	74.180 ± 0.436	0.873
8PA	76.670 ± 0.197	76.080 ± 0.492	76.550 ± 0.458	0.399
12PA	79.120 ± 0.214	78.900 ± 0.296	79.930 ± 0.438	0.147
4BSL	11.523 ± 0.178	11.425 ± 0.063	11.533 ± 0.090	0.919
8BSL	16.250 ± 0.061	16.206 ± 0.088	16.171 ± 0.136	0.821
12BSL	19.745 ± 0.068	19.728 ± 0.095	19.801 ± 0.130	0.908
4PB	5.172 ± 0.039	5.165 ± 0.035	5.196 ± 0.059	0.939
8PB	6.904 ± 0.050	6.869 ± 0.045	6.834 ± 0.066	0.750
12PB	8.653 ± 0.045	8.604 ± 0.064	8.791 ± 0.083	0.241

CC (*n* = 509), CT (*n* = 234), TT (*n* = 117). Mean ± SE. The numerals 0, 4, 8, and 12 indicate age in weeks. SL, shank length; SG, shank girth, CD, chest depth; CB, chest breadth; BBL, breast-bone length, PA, pectoral angle; BSL, body slanting length, PB, pelvis breadth. The unit of PA is °, while the units of all other indicators are cm.

**Table 3:** Genotypic effects of the *pre-miR-1453* (rs16159272) variant on serum biochemical parameters.

Indices	Genotype			*p*
	CC	CT	TT	
ALT	1.970 ± 0.126	1.820 ± 0.160	1.730 ± 0.212	0.604
AST	286.250 ± 3.284	284.340 ± 4.773	280.330 ± 7.430	0.736
γ-GT	15.610 ± 0.309	15.790 ± 0.420	14.580 ± 0.681	0.268
AKP	680.770 ± 25.099	731.260 ± 35.592	768.120 ± 59.595	0.248
TP	43.144 ± 0.437 a	42.741 ± 0.612 b	43.972 ± 0.785 a	0.008 **
ALB	16.760 ± 0.117 a	16.473 ± 0.170 b	16.940 ± 0.234 a	0.042 *
GLOB	26.561 ± 0.368 a	26.345 ± 0.528 b	27.084 ± 0.676 a	0.019 *
CHE	1.916 ± 0.027 a	1.841 ± 0.036 b	2.010 ± 0.061 a	0.005 **
CRE	3.780 ± 0.366	3.420 ± 0.535	4.230 ± 0.920	0.703
GLU	8.638 ± 0.188	9.286 ± 0.298	8.305 ± 0.321	0.070
TC	3.156 ± 0.037	3.154 ± 0.064	3.122 ± 0.084	0.927
TG	0.420 ± 0.005	0.403 ± 0.008	0.401 ± 0.011	0.110
HDL	1.995 ± 0.021	1.963 ± 0.033	1.958 ± 0.048	0.591
LDL	1.017 ± 0.022	1.015 ± 0.035	1.066 ± 0.049	0.615
CPK	7441.21 ± 102.34 a	7402.72 ± 142.62 b	6716.20 ± 222.74 c	0.008 **
LDH	2822.20 ± 24.64 a	2740.55 ± 43.41 b	2717.54 ± 45.70 a	0.042 *
AMY	425.43 ± 11.16	445.79 ± 15.21	418.36 ± 19.70	0.486

CC (*n* = 509), CT (*n* = 234), TT (*n* = 117). Mean ± SE. ALT, AST, γ-GT, AKP, CPK, LDH, and AMY were measured in U/L; TP, ALB, and GLOB were measured in g/L; CHE was measured in kU/L; CRE was measured in μmol/L; GLU, TC, TG, HDL, and LDL were measured in mmol/L. Significance notations are as defined in Table 1.

## Data Availability

The raw data of the Gushi–Anka F_2_ resource population used in this study are not publicly available, as this resource population is part of an ongoing research project involving multiple unpublished studies and pending intellectual property protection (including patent applications under substantive examination). Data are available from the corresponding author upon reasonable request, subject to confidentiality and intellectual property agreements.
